# HIV-Prevalence Mapping Using Small Area Estimation in Kenya, Tanzania, and Mozambique at the First Sub-National Level

**DOI:** 10.5334/aogh.3345

**Published:** 2021-09-27

**Authors:** Enrique M. Saldarriaga

**Affiliations:** 1 The Comparative Health Outcomes, Policy & Economics (CHOICE) Institute, University of Washington, Seattle, WA, US

## Abstract

**Background:**

Local estimates of HIV-prevalence provide information that can be used to target interventions and consequently increase the efficiency of resources. This enhanced allocation can lead to better health outcomes, including the control of the disease spread, and for more people.

**Methods:**

In this study, we used the DHS data phase V to estimate HIV prevalence at the first-subnational level in Kenya, Tanzania, and Mozambique. We fitted the data to a spatial random effect intrinsic conditional autoregressive (ICAR) model to smooth the outcome. Further, we used a sampling specification from a multistage cluster design.

**Results:**

We found that Nyanza (*P_i_* = 13.6%) and Nairobi (*P_i_* = 7.1%) in Kenya, Iringa (*P_i_* = 15.4%) and Mbeya (*P_i_* = 9.3%) in Tanzania, and Gaza (*P_i_* = 15.2%) and Maputo City (*P_i_* = 12.9%) in Mozambique are the regions with the highest prevalence of HIV, within country. Our results are based on publicly available data that through statistically rigorous methods, allowed us to obtain an accurate visual representation of the HIV prevalence at a regional level.

**Conclusions:**

These results can help in identification and targeting of high-prevalent regions to increase the supply of healthcare services to reduce the spread of the disease and increase the health quality of people living with HIV.

## Introduction

One of the most important challenges for Eastern and Southern Africa to achieve the 90-90-90 HIV targets have been initiation and retention in care [1]. The lack of resources to provide appropriate care to all people living with HIV (PLWHIV) limits the proportion of people that can achieve viral suppression which in turn increases the opportunity for disease spread [2,3]. Thus, it is essential to find ways to increase resources’ efficiency, to obtain the best possible health outcomes at the lowest investment. One way to achieve this is by improving targeting of interventions across geographical areas [4], which requires reliable information at the local level to guide policy decisions. In the context of initiation and retention to care, observing the prevalence – defined as the proportion of HIV positive people in a region geographically, demographically, and temporally defined – at a sub-national level could provide means to guide resource-allocation decisions.

The main issue with prevalence mapping is that estimations across sub-national regions are needed to obtain better information but are usually based on incomplete, local, survey data. Therefore, if no cases are recorded in a particular region, the *empirical* prevalence would be zero, even though that is likely erroneous. Hence, we recognize information is incomplete (i.e., not all cases have been recorded), and the modeled probability does not reflect the total population at-risk. In addition, because no completely-at-random sampling is feasible, survey data carries selection bias in its estimations. To correct these issues, we conduct prevalence mapping using small area estimation (SAE) [5,6].

The aim of this study was to estimate the prevalence in a sub-set of Eastern African countries at the first sub-national level, to create information relevant to locally focused policy decision- making.

## Methods

### Data

We used the DHS HIV and geospatial data [7]. The selected sub-set of countries are neighboring countries in Eastern Africa, have important differences in their national HIV prevalence estimates and have HIV and geospatial information in the same phase of the DHS Program for sampling design and data collection consistency. We decided for: Kenya, Tanzania, and Mozambique, whose estimated national prevalence for the 15 to 49 year-old population was 5.66%, 3.97%, and 11.98%, respectively in 2017 [8,9]. This is the most heterogeneous cluster, in terms of national HIV-prevalence, in East Africa that we were able to find. These countries have spatial and HIV data collected in the phase DHS-V, executed between 2007 and 2009. The prevalence mapping was conducted over the first administrative sub-national areas: regions within countries.

The geospatial information for each country was originally formed by the sampling cluster information and their associated boundaries. The former included the spatial points for all clusters within regions, while the latter included polygons for each region within a country. Using the coordinates of both datasets we identified to which region each cluster belonged. Then, we merged the information. The HIV data were geographically identified only by clusters. We used the merged spatial information to determine to which region each of the clusters in the HIV data belonged to.

The final spatial dataset is the appended data of the three countries containing the polygons for each of the sub-national regions. The final HIV dataset was also the appended data of the three countries containing the individual health outcomes (i.e., HIV positive or negative), the cluster, and the associated sub-national region.

### Model

Considering that the DHS survey used a multistage cluster design sampling, we conducted a SAE, with a spatial random effect intrinsic conditional autoregressive (ICAR) model [10] – the BYM model –, for a binary outcome. The random effects model has a higher precision than the direct estimation that just considers the sampling design [11]. The BYM model allows for indirect estimation utilizing the information across regions, based on defined neighbors, to perform the smoothing. A neighbor is a region with a single- or multiple-point shared border. We allow places with extra neighbors to have a higher influence on the results – the “B” style. Since we treated the sub-set of countries as a unit, the neighbor determination is not constrained within the country. Hence, the prevalence estimation *borrows* information from neighboring regions, even when these neighbors are in different countries.

To estimate the spatially smoothed prevalence, *p_i_*, in each region in the first sub-national level, denoted by *i*, we fitted the data to the following model: [12]

\begin{array}{*{20}{c}}
{logit\left( {{p_i}} \right)\ \sim \ N\left( {{\theta _i},{V_i}} \right)}\\
{{\theta _i} = {\beta _0} + {S_i} + {{\epsilon}_i}}\\
{{S_i}\  \sim ICAR\left( {\sigma _S^2} \right)}\\
{{{\epsilon}_i}{{\sim}_{iid}}N\left( {0,\;\sigma _{\epsilon}^2} \right)}
\end{array}

Where *p_i_* is the direct (design-based weighted) estimate of the prevalence for the region *i*, modeled using a logit-link given the binary nature of the outcome; *V_i_* is the estimated variance for each region; *β_0_* is the intercept which represents the shared, baseline effects; *S_i_* the spatial smoothing; and *ϵ_i_* is the unstructured random effects. This model does not include covariates. We perform the analysis using the SUMMER package [13] in R.

## Ethics

This is a secondary data analysis that used publicly available information and had no contact with the participants. As such, it was deemed not human research by the IRB of the University of Washington and no level of IRB-review was necessary.

## Results

The original dataset, aggregated for the three countries had a sample size of 39,575 observation (Kenya, n = 7,001; Tanzania, n = 15,597; Mozambique, n = 16,976). After deleting the observation containing missing data in the geographic information only (no missing values in the HIV data was found), the final sample size was 39,258 (99.2%). Given the small number of missed observations, we did not perform a missing-at-random analysis.

**Figure 1:**
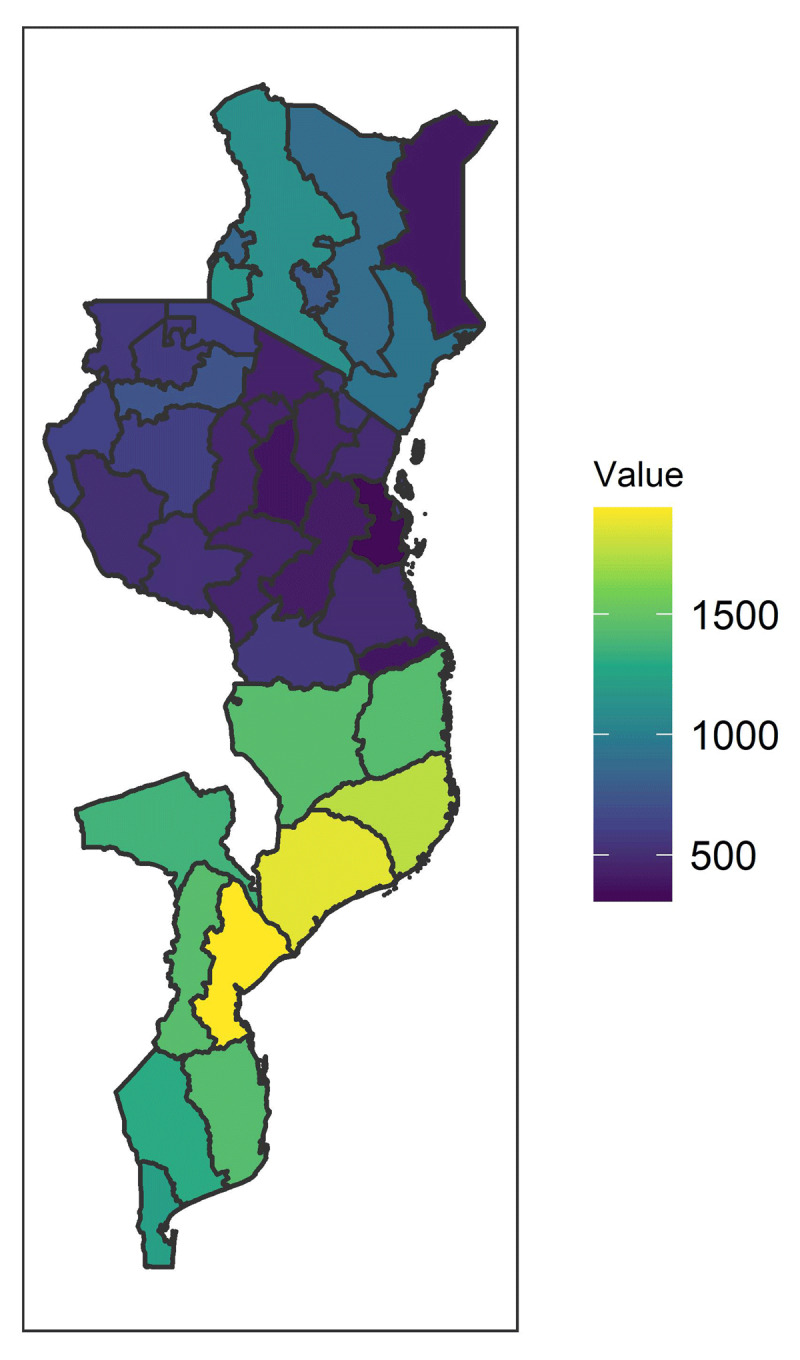
Regions under analysis color-coded by their sample size.

We found 45 regions in total. The sample size varies highly across these regions, ranging from 308 to 1,942 observations (***Figure 1***). Tanzania was the country with the highest number of regions, 26, and at the same time the smallest sample size for each one. Kenya, with 8 regions, and moreover Mozambique, with 11 regions, have higher associated sample size.

**Figure 2:**
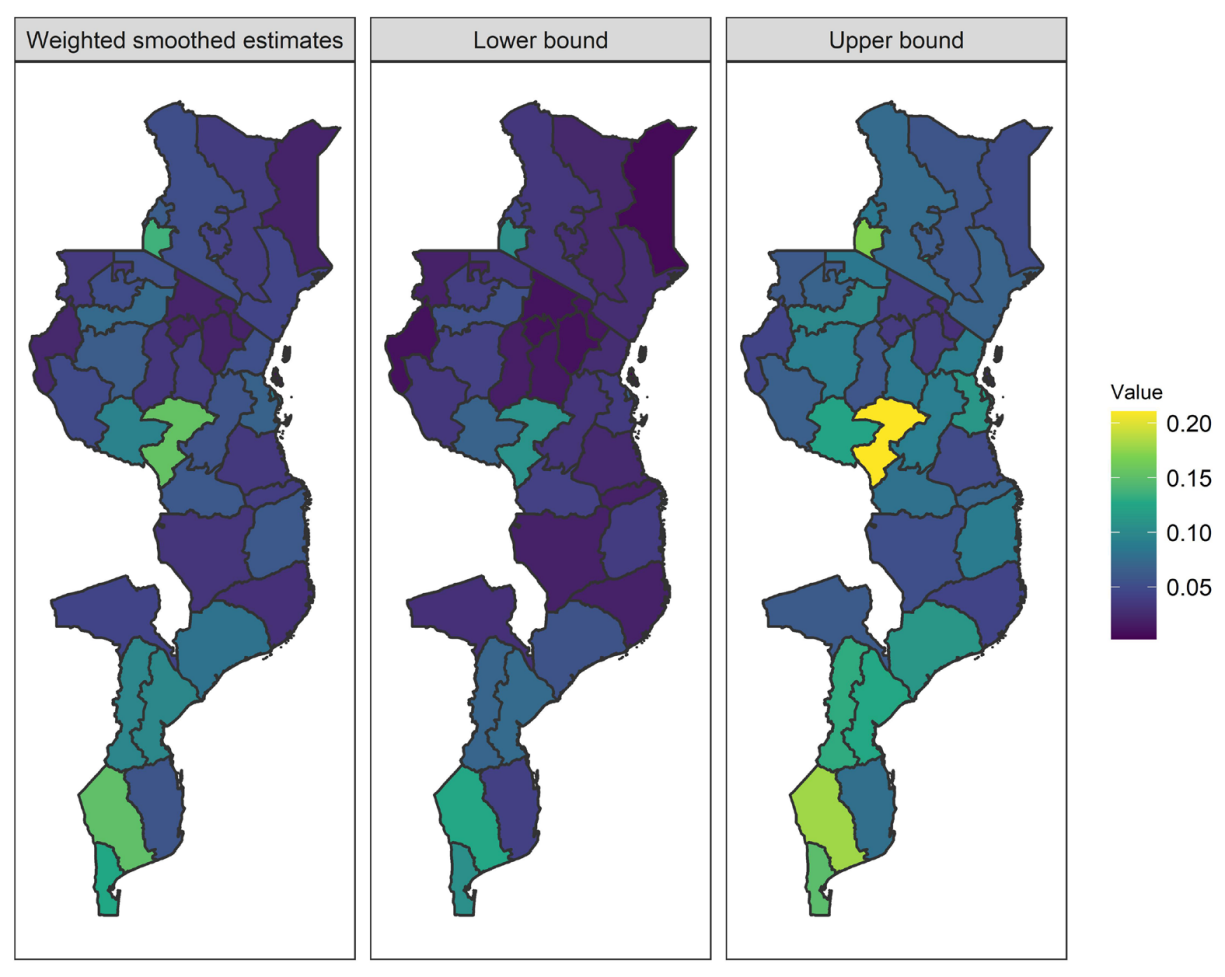
Maps of point estimate and 95% credible intervals for the posterior mean of the weighted smoothed model.

**Figure 3:**
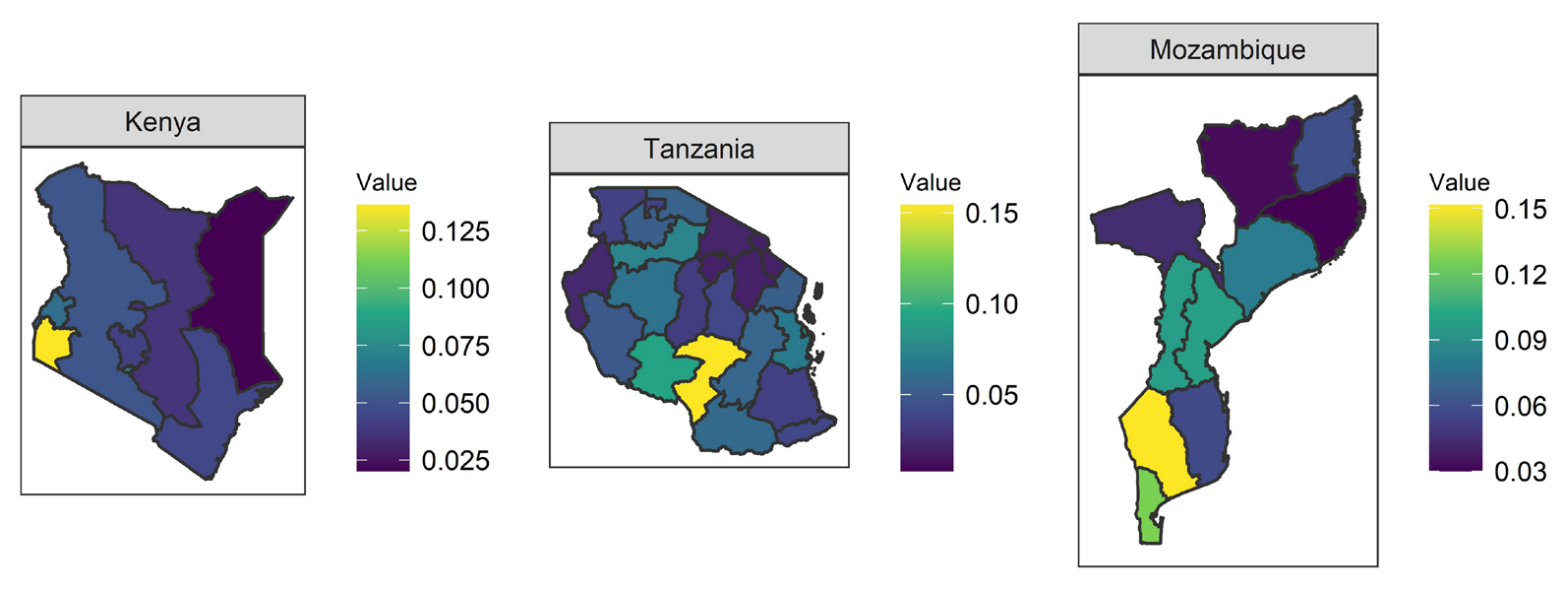
Zoom in the HIV-prevalence maps for each country. Kenya in the left, Tanzania in the middle, and Mozambique in the right panel.

***Figure 2*** shows the results of the SAE. The estimated prevalence for all regions ranged from 7.24% to 15.5%; with a median of 5.13% and a standard deviation (SD) of 0.38. ***Figure 3*** shows the point-estimate results for each country. The scale for the color-code is independent for each country in ***Figure 3***.

Our analysis allowed us to identify the three regions with the highest prevalence in each country. Nyanza (*p_i_* = 13.6%), Nairobi (*p_i_* = 7.1%), and Western (*p_i_* = 6.3%), in Kenya. In Tanzania, Iringa (*p_i_* = 15.4%), Mbeya (*p_i_* = 9.3%), and Dar es Salaam (*p_i_* = 9.1%). Finally, in Mozambique, Gaza (*p_i_* = 15.2%), Maputo City (*p_i_* = 12.9%), and Maputo (region) (*p_i_* = 12.7%). The Supplemental Material contains the estimated prevalence and 95% credible intervals for each region. In Kenya and Tanzania, we can observe that the highest prevalence is almost an outlier compared to the rest of the country. These results warrant a prioritization of these regions for HIV prevention and treatment health services.

## Discussion

We conducted a HIV-prevalence mapping for a subset of east African countries, with a SAE estimation using a BYM model. This approximation has multiple theoretical benefits. First, the SAE is consistent with the small sample size that observed in many of the areas under estimation. Second, our model acknowledges the sampling design and the associated distribution of sampling probabilities. Third, the spatial smoothing process allowed the model to borrow information from the neighbors in the estimation of each posterior to improve the precision of the estimates, which is particularly helpful in the presence of sparse data such as the DHS dataset. Further, our study has an additional advantage because it defines the neighbors beyond the country borders, by taking the sub-set of countries as a unit. We believe this was particularly useful in the estimation of the prevalence for Tanzania, where all its regions had a small sample size.

**Figure 4:**
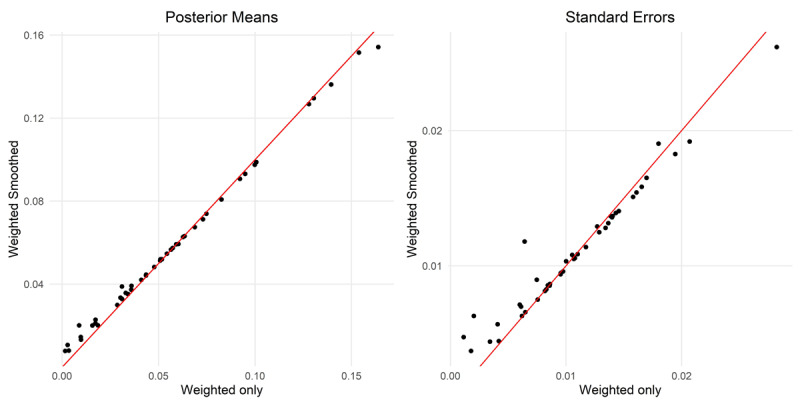
Comparison of estimated prevalence (left panel) and standard errors (right panel) between the weighted only and weighted and smoothed SAE estimates.

We examined the difference in performance for the estimation of prevalence at the first-subnational level, for the weighted model that includes a correction for cluster sampling, and the weighted-smooth model that includes a smoothing process for the outcome. ***Figure 4*** shows that the smoothing process pulled the posterior estimates towards the middle of the distribution, increasing the value of the estimates in the lower half, and reducing it for the estimates in the higher half. The right-side panel shows that the standard errors created by the smoothing analysis tend to be lower in comparison, denoting estimates with higher precision. Thus, because the smoothing model estimates each prevalence using the information provided by its neighbors, the resulting distribution of prevalence for all regions is less disperse, with the extreme values pulled towards the middle.

Regarding the spatial weights for the matrix of neighbors, “B” and “W” are the most used styles [14]. In essence, under the “B” style, regions with more neighbors would have a higher influence on the results, while under “W” having extra neighbors do not affect the region’s weight. Thus, the “B” style is a better option when migration patterns and likelihood of contact plays a relevant role in the outcome under analysis, as it is the case of HIV transmission [15]. Nevertheless, we performed separate analyses with both styles obtaining the same results for the posteriors and the standard deviation. We decided in favor of the “B” style given its theoretical advantages over the alternative.

**Figure 5:**
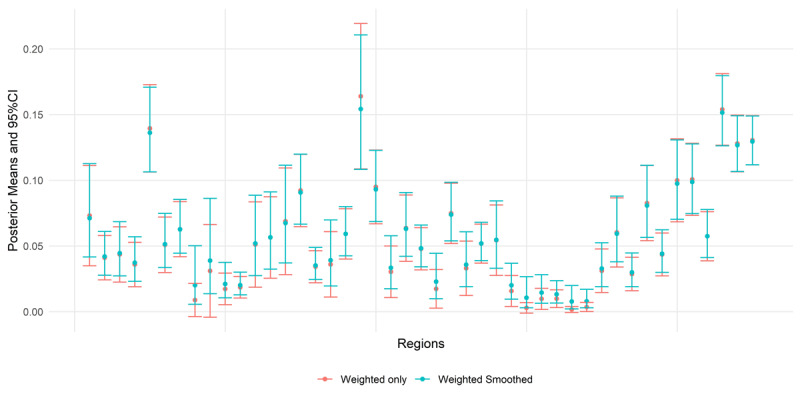
Error bars for the weighted smoothed SAE estimates and the naïve (direct) ones.

According to ***Figure 5***, even though the weighted-only model includes a correction for the sampling methodology, the resulting estimates for 4 regions are still not significantly different from zero; i.e., the 95% credibility intervals of these estimates include zero. This demonstrates an important advantage of the smoothing process: by shrinking the estimates towards the mean, it eliminates prevalence estimates non-statically different from zero. Further, it exemplifies the difference between the observed and the empirical prevalence [6]. The lack of cases in a cluster does not mean that there are no cases in that geographical area. Hence, surrogating the estimated risk from adjacent areas is imperative to estimate a prevalence closer to the *real* risk of infection for HIV.

Our estimates are systematically lower compared to previous analysis that have mapped the prevalence of HIV in sub-Sharan Africa [16], which is due to the granularity of the data used for the analysis. Dwyer-Lindgren et al. collected 38,897 data points in 134 seroprevalence survey and sentinel surveillance of antenatal care clinics for a total of 46 countries. Further, they gathered information from a 17-year period. Given this, the analytical process can smooth the estimates across geographic and temporal units and provide better estimates. Additionally, Dwyer-Lindgren et al calibrated the model using national estimations from the Global Burden of Disease [17], which provides an additional level of external validity but also introduces more bias in the analysis. Nonetheless, the estimated credibility intervals for both studies overlap in all cases for which a comparison was possible, and more importantly, the results of both studies are consistent regarding which regions within countries have the highest prevalence.

The objective of this study was to present reliable, local prevalence information to inform policy decision in the context of optimize resource allocation. In that regard, the temporality of the data is not as important since the absolute numbers can change but the relative risk is less likely to change over time. Second, our methodology is in concordance with both the nature of the data – by including a correction due to the sampling design – and the epidemiology rationale – by using a smoothing process to limit the probability of having prevalence equal to zero and modeling the outcome in each region as part of a bigger area, rather than in isolation.

This study is not without limitations. First, the data that we used were collected between 2007 and 2009. Hence, the picture that our estimates present is likely outdated considering how fast the HIV epidemic variables move over time. We chose the phase DHS-V because that was the only phase in which our sub-set of countries had both HIV and geospatial information. Second, although socioeconomic and demographic variables influence the prevalence of HIV [18,19] and these variables are likely spatial correlated, our model did not include covariates. The main reason was the lack of an individual identifier between the HIV data and other DHS surveys to match the datasets.

## Conclusions

Our results are based on statistically appropriate methods that allowed us to obtain an accurate visual representation of the HIV prevalence in the subset of African countries we chose. These results can help in identification and targeting of high-prevalent regions to increase the supply of healthcare services to reduce the spread of the disease and increase the health quality of PLWHIV.

This study builds on secondary publicly available data and generates reliable estimates that can be used to target interventions. This creates an important opportunity to apply the same methodology in other settings, where data might be scarce and resources to supplement data collection unavailable.

## Additional File

The additional file for this article can be found as follows:

10.5334/aogh.3345.s1 Supplemental Material. The Supplemental Material contains the estimated prevalence and 95% credible intervals for each region in the three countries analyzed: Kenya, Tanzania, and Mozambique.
